# A ruptured mesenteric lymphangioma causing peritonitis: a case report

**DOI:** 10.1093/jscr/rjae319

**Published:** 2024-05-18

**Authors:** Ayoub Kharkhach, Chafik Rhoul, Andrea Police, Andrea Mabilia

**Affiliations:** Department of General Surgery, Faculty of Medicine and Pharmacy, Mohammed First University, Oujda 60000, Morocco; Department of Visceral and Digestive Surgery, Simone Veil Hospital, Eaubonne 95600, France; Department of General Surgery, Faculty of Medicine and Pharmacy, Mohammed First University, Oujda 60000, Morocco; Department of Visceral and Digestive Surgery, Simone Veil Hospital, Eaubonne 95600, France; Department of Visceral and Digestive Surgery, Simone Veil Hospital, Eaubonne 95600, France; Department of Visceral and Digestive Surgery, Simone Veil Hospital, Eaubonne 95600, France

**Keywords:** cystic lymphangioma, small bowel mesentery, peritonitis, open surgery

## Abstract

Cystic lymphangioma is a rare disease that is mainly diagnosed in childhood. When diagnosed, the lesion presents an indication for surgery due to the risk of serious complications. Herein, we report the case of a 32-year-old patient who presented to the emergency room for abdominal pain that developed 2 days before with worsening symptoms and abdominal pain in the last 24 hr. The computed tomography showed diffuse wall thickening of the jejunum and proximal ileum with mesenteric fat infiltration, a mesenteric collection, and a moderate volume of ascites extending into the pelvis. A laparotomy was performed, revealing diffuse chemical peritonitis with a crater-like lesion in the jejunal mesentery, secreting lymphatic fluid. The mesenteric lesion was then excised, and the histological examination showed a ruptured cystic lymphangioma. Lymphangiomas of the small bowel mesentery are rare and may be exceptionally associated with bowel occlusion or peritonitis.

## Introduction

Mesenteric cystic lymphangiomas (MCLs) recognized as benign vascular congenital tumors, are a rarity, predominantly found in extrabdominal localization in children [1]. However, lymphangiomas in the peritoneal cavity are extremely rare, particularly in adults, and 65% of MCLs are diagnosed before the age of 2 years old [2]. They represent 5%–6% of pediatric benign tumors and display a notable male predilection [3, 4]. The etiology of MCLs is not completely clear. It may be related to an abnormal embryonic development of the lymphatic system in the pediatric age group [3]. The clinical symptomatology of MCLs is polymorphous and not specific ranging from incidentally discovered lesions to acute abdominal pain. Ultrasound (US), computed tomography (CT), and magnetic resonance imaging are the radiological techniques used in the evaluation of MCLs, yet the histological confirmation constitutes an imperative component of diagnostics [3, 5, 6]. Surgery is the best curative treatment with complete excision, even when the patient is asymptomatic, to avoid any recurrence or complications [4, 5]. This report highlights a unique case of an adult presenting with a ruptured mesenteric lymphangioma, resulting in acute peritonitis.

## Case report

**Figure 1:**
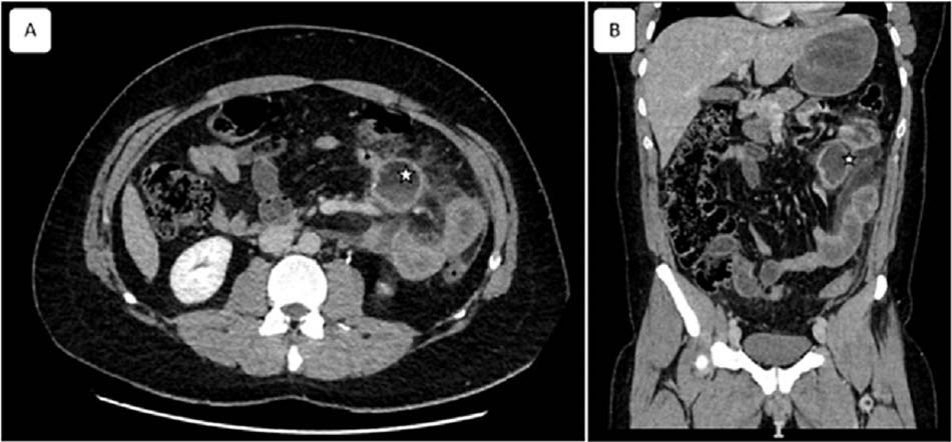
Coronal and sagittal views of computed tomography showing a mesenteric collection of 51 × 33 with mesenteric fat infiltration (asterisk) related to the peritonitis caused by the ruptured cyst. Reconstructed images: (A) axial and (B) sagittal view.

**Figure 2:**
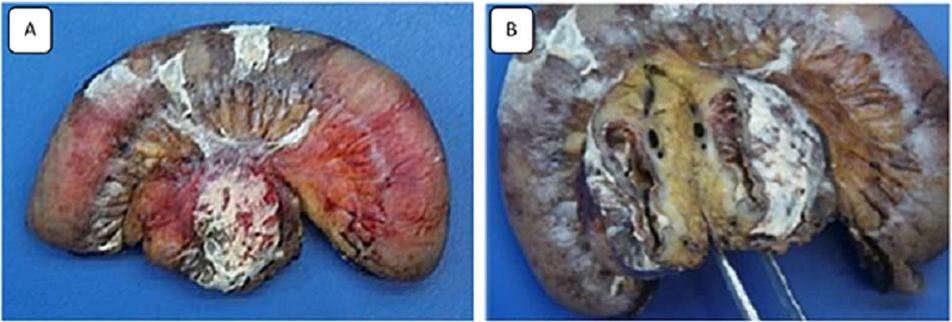
Images of the surgical specimen showing the monobloc resection of the proximal jejunum and the perforated mesenteric cystic lymphangioma.

A 32-year-old patient without medical history was admitted to the emergency department for epigastric pain syndrome, which developed 2 days before with worsening symptoms in the last 12 hr. On clinical examination, the patient had no fever and was hemodynamically stable. The abdomen was distended, with widespread pain at palpation. On blood tests, there were stable hemoglobin at 15.9 g/100 ml, a mild inflammatory syndrome with elevated white blood cells at 13.900/mm^3^ and C reactive protein at 33 mg/L. The CT examination showed diffuse wall thickening of the jejunum and proximal ileum with mesenteric fat infiltration, a mesenteric collection of 51 × 33 mm, and a moderate volume of ascites extending into the pelvis (Fig. 1). A laparotomy was performed, revealing diffuse chemical peritonitis with aspiration of 1 L of chylous ascites. A whitish crater-like lesion with lymphatic fluid outlet was found in the jejunal mesentery, ~50 cm distal to Treitz’s ligament. A monobloc resection of the mesenteric lesion with the affected jejunal segment (Fig. 2) and a manual termino-terminal anastomosis were performed. Postoperatively, an empirical antibiotic therapy (amoxicilline—clavulanic acid 3 g/d, and Gentamicin 5 mg/kg/d) was administered for 3 days only, since the intraoperative bacteriological examination was negative. The abdominal drain was removed after two postoperative days. The patient was discharged without complications on the sixth postoperative day.

The specimen consisted of one piece of bowel resection measuring 25 × 3.5 cm. Macroscopically, the mesentery adjacent to the small bowels contained a 4 × 4 cm ruptured unilocular cyst with a thick wall, but no vegetation or solid component were found. Microscopically, the sections showed numerous dilated lymphatics throughout the cyst wall, containing protein material, and lined with a layer of flattened normal cells. The adjacent serosa of the cyst was covered with a fibrinoleukocytic coating associated with opalescent macrophages. Immunohistochemical stains were positive for D2-40 and highlighted many lymphatics, defining the diagnosis of cystic lymphangioma.

The patient had an uneventful postoperative course and no evidence of recurrence 6 months after the operation.

## Discussion

Intra-abdominal mesenteric lymphangiomas are uncommon benign tumors, usually occurring in childhood. They represent <1% of all lymphangiomas [6]. During embryogenesis, the peripheral lymphatic system develops from the primitive venous system [7]. A well-established theory suggests that lymphangiomas result from a lack of connection between a secondary lymphatic bud and the venous system [2, 7].

Cystic lymphangiomas are considered malformative vascular tumors, for which no malignancy potential has yet been demonstrated. The acquired or iatrogenic origin of lymphangiomas was also evoked as a late secondary formation related to an obstruction of the lymphatic vessels following inflammation, abdominal trauma, surgery, or radiotherapy [8].

The most common symptom of cystic lymphangiomas is abdominal pain, swelling or palpable mass, and sometimes lower limb lymphedema [7, 9]. Other uncommon complications with acute symptomatology have been reported in the literature, such as intracystic hemorrhage or infection and digestive hemorrhage [8]. In our case, a spontaneous rupture of the cyst was supposed to occur, which is a possible but extremely rare complication. Furthermore, the disseminated peritoneal cystic lymphangiomatosis is a rare form of the disease. It might mimic peritoneal carcinosis, and it is usually associated with mediastinal lesions and chylothorax [7, 10]. Abdominal US, CT, and exploratory laparoscopy may be helpful in establishing the diagnosis [11]. Even though, preoperative diagnosis is only possible in 22.6% of cases [12]. On CT imaging, the lesions appear as a uni- or multilocular cystic mass with contrast enhancement of the wall and septum [10, 13]. Despite the rare incidence, these lesions could be accurately diagnosed with an US-guided fine needle aspiration endoscopy (EUS-FNA). Aspiration usually draws out a milky-white fluid containing a high concentration of lymphocytes and triglycerides (>500 mg/dl) [14].

In addition to the useful role of imaging, the definitive diagnosis is confirmed by histopathology after surgical resection [7, 9]. Three forms of lymphangiomas are described: the capillary lymphangioma, the lymphangioma cavernosum, and the cystic lymphangioma [9, 10]. In our case, histopathology confirmed cystic lymphangioma. This type is characterized by lymphatic spaces of various sizes that contain smooth muscle fascicles and collagen. Cystic lymphangioma is not always clearly differentiated from the cavernous type; indeed, both can contain cavernous areas [9]. The diagnosis of cystic lymphangioma is confirmed using three standard histological criteria: cyst lined by a flat endothelial epithelium, small lymphatic spaces, and abundant lymphoid tissue [6, 13]. The immunohistochemical analysis for the endothelium markers CD31 and CD34 shows diffuse positivity of the cyst endothelium [13]. In our case, the histological diagnosis was particularly complicated due to the inflammation and necrosis present in the tissues, and the final diagnosis was achieved by the immunohistochemical study of D2-40.

The gold standard treatment for cystic lymphangioma is the total surgical excision. Open or laparoscopic approach it is equivalent. If feasible, the excision must be as complete as possible to minimize the risk of recurrences, remaining conservative with no major sacrifice of adjacent organs, given the benign nature of lymphangioma. However, segmental bowel resection is necessary when the enucleation is not possible [3, 12]. Recently, alternatives to surgery for unresectable diseases have been proposed. These include percutaneous intracystic injection of sclerosing agents, such as alcohol or OK-432 [10, 15]. OK-432 is made of freeze-dried streptococcal preparation, which induces a strong local inflammatory reaction. However, long-term outcomes showed high rates of recurrences, up to 100% in some series [15].

Differential diagnoses of an intra-abdominal cystic mass include enteric cyst, mesothelial cyst, pancreatic pseudocyst, cystic mesothelioma, spindle cell cystic tumor, and cystic teratoma [12].

## Conclusion

Cystic lymphangioma are rare and benign tumors of the lymphatic system, mainly diagnosed in childhood. The clinical presentation is polymorphic, and complications, such as rupture and peritonitis, are extremely rare. The diagnosis is suspected by imaging and can only be confirmed by histological examination of the surgical specimen. In case of a non-symptomatic injury, monitoring is the rule. In case of symptomatic injury, complete removal of the lesion is the best option to avoid recurrence.
